# The probabilistic pool punishment proportional to the difference of payoff outperforms previous pool and peer punishment

**DOI:** 10.1038/s41598-022-10582-5

**Published:** 2022-04-22

**Authors:** Tetsushi Ohdaira

**Affiliations:** Institute of Information and Media, Aoyama Gakuin University, 5-10-1 Fuchinobe, Chuo-ku, Sagamihara-city, Kanagawa 252-5258 Japan

**Keywords:** Social evolution, Computational science

## Abstract

The public goods game is a multiplayer version of the prisoner’s dilemma game. In the public goods game, punishment on defectors is necessary to encourage cooperation. There are two types of punishment: peer punishment and pool punishment. Comparing pool punishment with peer punishment, pool punishment is disadvantageous in comparison with peer punishment because pool punishment incurs fixed costs especially if second-order free riders (those who invest in public goods but do not punish defectors) are not punished. In order to eliminate such a flaw of pool punishment, this study proposes the probabilistic pool punishment proportional to the difference of payoff. In the proposed pool punishment, each punisher pays the cost to the punishment pool with the probability proportional to the difference of payoff between his/her payoff and the average payoff of his/her opponents. Comparing the proposed pool punishment with previous pool and peer punishment, in pool punishment of previous studies, cooperators who do not punish defectors become dominant instead of pool punishers with fixed costs. However, in the proposed pool punishment, more punishers and less cooperators coexist, and such state is more robust against the invasion of defectors due to mutation than those of previous pool and peer punishment. The average payoff is also comparable to peer punishment of previous studies.

## Introduction

In the context of social dilemmas, it is widely known that a system of punishment is necessary for the evolution of cooperation. Punishment is a factor that encourages cooperation, however, those who punish others lose their fitness because the cost of punishment reduces their payoff. For this reason, whether punishment is really necessary for the evolution of cooperation, especially whether a system of punishment will evolve, is highly controversial. For example, some studies^1–7^ have a negative view of punishment, while others^8–18^ have a positive view of punishment.

In the previous studies that have shown a negative point of view, for example, in the prisoner’s dilemma game, Dreber et al.^2^ state that the introduction of peer punishment results in the reduction in the average payoff of the population as well as the introduction of pool punishment. Peer punishment is the one of two types of punishment. Peer punishers directly punish uncooperative opponents by paying some cost. Pool punishment is the other one. When pool punishers pay the investment, they also pay the cost to the punishment pool in advance. In the public goods game, which is a multiplayer version of the prisoner’s dilemma game, Rand and Nowak^5^ extend the public goods game to include the full set of punishment strategies and find that punishment no longer increases cooperation, and that natural selection favours substantial levels of antisocial punishment for a wide range of parameters. They also show that model predictions consistent with the results of behavioural experiments, and punishment is mostly a self-interested tool for protecting oneself against potential competitors. Nowak^6^ states that punishment is not a mechanism for the evolution of cooperation, but only complements other mechanisms such as indirect reciprocity, group selection, and network reciprocity. Wu et al.^7^ describe that punishment does not necessarily facilitate cooperation in one-to-one interactions such as the prisoner’s dilemma game.

In contrast to the preceding argument, regarding positive discussions, Traulsen et al.^12^ consider the public goods game with cooperators, defectors, cooperative punishers, and the players who abstain from public goods interactions. They show that cooperation (and punishment) is possible only if interactions are voluntary when mutation rates are small. Sigmund et al.^14^ compare the prevailing model of peer punishment with pool punishment. Pool punishment facilitates the sanction of second-order free-riders (who cooperate but do not sanction), because those free-riders can be distinguished even if everyone contributes to the common good. Garcia and Traulsen^16^ state that Rand and Nowak^5^ discuss very limited cases where players refrain from collective action, and show that cooperators who only punish defectors can thrive well when the abstainers are isolated even if players have the choice of antisocial behaviour. Perc and Szolnoki^17^ propose the adaptive punishment that allows players to change the degree to which they punish their opponents in proportion to the degree of success of cooperation. The adaptive punishment promotes the reciprocity based on spatial connections between players (below simply referred to as connections), and as a result, enhances cooperation. Nakamaru and Dieckmann^18^ investigate the evolution of social reaction norms and mainly show that the mechanism where evolution to enhanced cooperation and stricter punishment reaction norms reinforce each other works best in the case of severe punishment.

The discussion regarding punishment is given in more detail below. Regarding peer punishment in the public goods game, Helbing et al.^19^ show that the consideration of punishment enables us to understand the formation and development of cooperators who punish defectors. Szolnoki et al.^20^ study the impact of pool punishment on the spatial public goods game with cooperators, defectors, and pool punishers as three competitive strategies. Helbing et al.^19,21,22^ specifically compare the efficiency of pool punishment with that of peer punishment in maintaining social advantage. Chen et al.^23^ show that the introduction of punishment has a positive effect in the public goods game, especially for large group-sized cooperation, but is not optimal for medium group-sized cooperation. Sasaki et al.^24^ study deposits that will be refunded as long as committers adhere to the donation game and punish free-riders and non-comitters.

We discuss other studies regarding punishment as follows. Gardner and West^25^ state that individuals show greater cooperation when interacting with those who have the high possibility of punishing others. Egas and Riedl^26^ show that punishment is strongly dominated by its cost-to-impact ratio. Traulsen et al.^27^ present that the majority of subjects would choose pool punishment if second-order free-riders would also be punished. Schoenmakers et al.^28^ show that central agencies such as police can be crucial to the evolution of punishment. Perc^29^ shows that pool punishment in structured populations is sustainable, but it is limited to the case where second-order free riders are also sanctioned to the extent that they cannot prevail. Chen and Perc^30^ show that the optimal distribution of resources within the framework of institutional punishment depends on whether absolute or degree-normalized payoffs are used. Perc et al.^31^ systematically review the main results obtained in the area of statistical physics of human cooperation and state that the problem of the cost of punishment can be solved by probabilistically sharing responsibility for sanctioning defectors. In the spatial prisoner’s dilemma game, Ohdaira^32,33^ shows that cooperation evolves not only in different types of spatial structures but also in the case where both strategy and spatial structure evolve by changing the probability of punishment according to the difference of payoff between players.

The alternative discussions regarding punishment are as follows. Szolnoki and Perc^34^ consider traditional cooperators and defectors, as well as cooperators punishing defectors and defectors punishing cooperators. They show that antisocial punishment does not prevent cooperation if the synergistic effects are high enough to sustain cooperation based on the network reciprocity and is viable only if the synergistic effects are low, punishment is necessary for cooperation, and the cost-to-fine ratio is low. Chen and Szolnoki^35^ reveal that cooperators should pay special attention to the growing capacity of renewable resources depending sensitively on the fraction of cooperators and the total consumption of all players in addition to a delicately adjusted punishment. Lee et al.^36^ introduce a policelike or mercenary punisher who watches the population and punishes defectors and show that the maximal average outcome can be reached at an intermediate cost value of punishment.

Here, this study especially focuses on peer punishment and pool punishment in the public goods game. As described before, peer punishment means that a player pays the cost and directly imposes the punishment on a defector, and pool punishment means that a player pays the cost to the punishment pool in advance. The advantages and disadvantages of peer punishment and pool punishment are as follows. Peer punishment enables a player to punish a defector directly, but it has the disadvantage of high cost of punishment. On the contrary, pool punishment has a lower cost required for punishment than peer punishment, but it has the disadvantage that a punisher must pay the cost to the punishment pool and fixed costs are incurred especially if we do not consider the punishment on second-order free riders (those who invest in public goods but do not punish defectors).

In order to eliminate such a flaw of pool punishment, this study proposes the probabilistic pool punishment proportional to the difference of payoff. In the proposed pool punishment, pool punisher compares his/her payoff with the average payoff of the opponent public goods game participants and pays the cost to the punishment pool only if he/she is disadvantageous in terms of payoff. This study considers the dynamics of four types of players, i.e., punishers (contributing public goods and punishing defectors), defectors, cooperators (only contributing public goods), and non-participants in the public goods game on the regular and the random one-dimensional lattice with the average degree < *k* >  = 4. Then, we compare the proposed pool punishment with peer and pool punishment of previous studies to investigate whether the flaw of pool punishment already mentioned can be eliminated.

## Model

The public goods game of this study is based on the framework by Traulsen et al.^12^ where investment in public goods is distributed to all participants, including the invested player. When the number of participants in the game is *n*, the number of cooperators is *N*_*c*_, and the investment of cooperators in public goods is *c*, cooperators will have the payoff of (*rcN*_*c*_/*n*)-*c*, while defectors will gain the payoff of *rcN*_*c*_/*n*. The value *r* is a factor multiplying all summed-up contributions, and the best response of the participants is defection (not investing in public goods). Therefore, in the public goods game, punishment on defectors is necessary to encourage cooperation. As described before, there are two types of punishment: peer punishment and pool punishment^14^. Peer punishers pay the investment *c* in public goods and then impose the punishment *b* on the defectors in the group by paying the cost *g*. Therefore, when there are *Ny* defectors and *Nw* peer punishers in the group, defectors will be punished with a sum of *bNw*, and peer punishers will bear the cost *gNy*. On the other hand, when pool punishers pay the investment *c*, they also pay the cost *G* to the punishment pool in advance. Defectors will be fined the punishment *BNv* proportional to the number of pool punishers, *Nv*.

**Figure 1:**
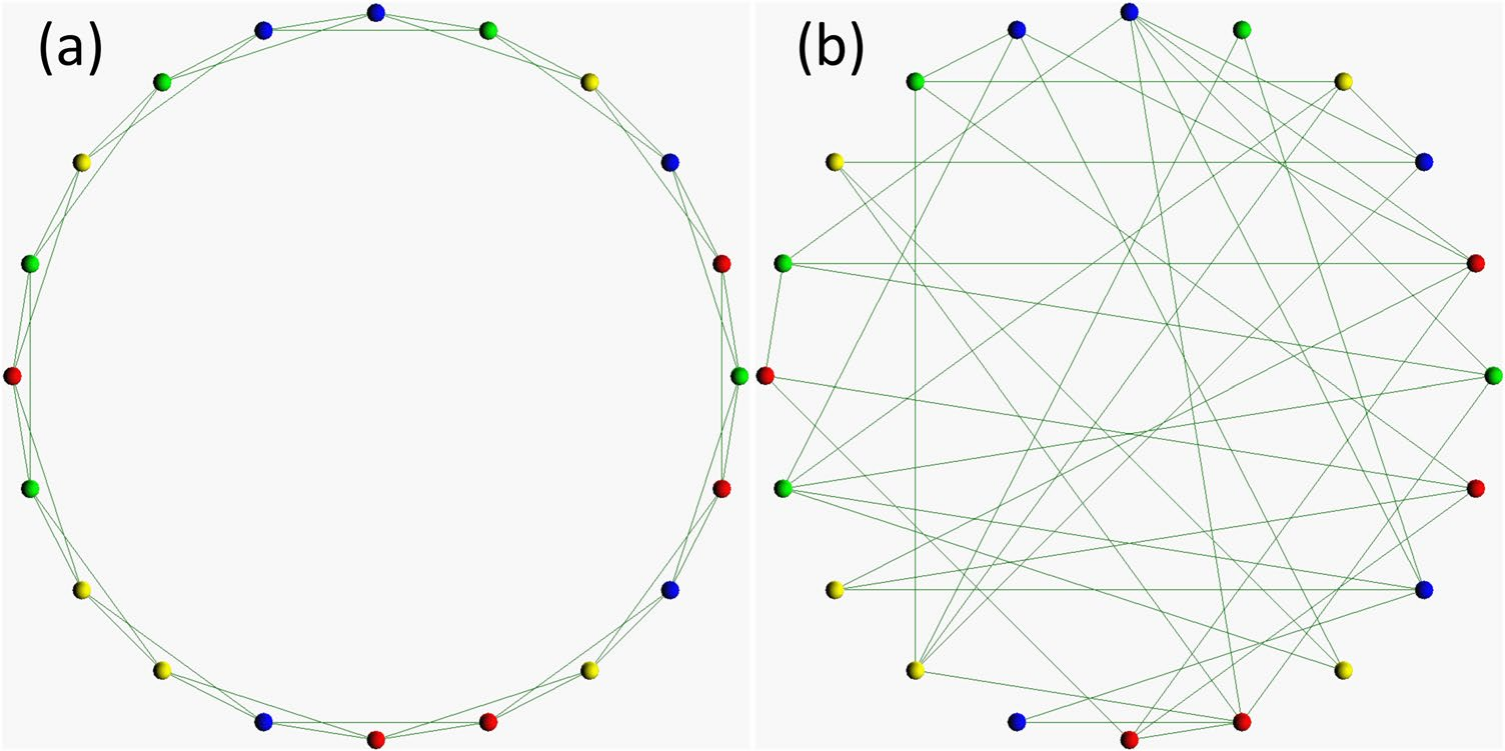
The sample spatial structure of (**a**) the regular and (**b**) the random one-dimensional lattice. The number of players is 1000 in the actual simulations, but it is 20 in this figure. The meaning of each colour is as follows: punishers are shown in blue, defectors are in red, cooperators are in green, and loners are in yellow.

Here, we consider the dynamics of four types of players, i.e., punishers (contributing public goods and punishing defectors), defectors, cooperators (only contributing public goods), and non-participants (below referred to as loners) in the public goods game on the regular and the random one-dimensional lattice with the average degree < *k* >  = 4. Figure 1a,b shows the sample spatial structure of the regular and the random one-dimensional lattice. A vertex shows a player, and opponent players of each player in public goods game interactions are defined by edges. Note that this figure has only 20 players so that we can easily grasp each spatial structure. The detail of how to construct each spatial structure is described in the previous study of the author^32^. To ensure that the group of pool punishers or peer punishers outperforms the group of loners in terms of payoff, the payoff of loners (*σ*) should be smaller than (*r* − 1)*c*-*G* in the case of pool punishment, or (*r* − 1)*c* in the case of peer punishment.

**Figure 2:**
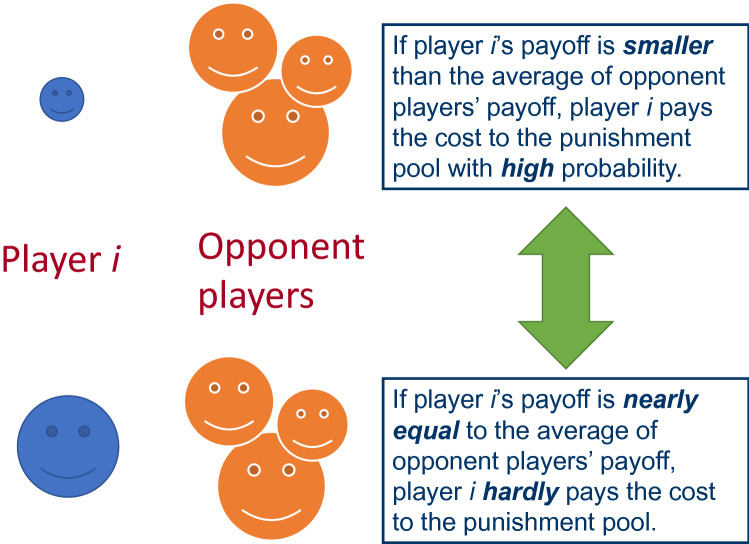
This figure illustrates the characteristics of the probabilistic pool punishment proportional to the difference of payoff.

As described before, pool punishment is disadvantageous in comparison with peer punishment because pool punishment incurs fixed costs especially if second-order free riders (those who invest in public goods but do not punish defectors) are not punished. In order to eliminate such a flaw of pool punishment, this study proposes the probabilistic pool punishment proportional to the difference of payoff. In the proposed pool punishment, when the payoff of player *i* is $P_{i}$ and the average payoff of the players with connections to player *i* is $\overline{{P_{i} }}$, player *i* pays the cost *G* to the punishment pool with the probability $q_{i}$ = ($\overline{{P_{i} }} - P_{i}$) / $P_{i}$ ($P_{i}$ > 0). If $\overline{{P_{i} }}$ is equal to or larger than $2P_{i}$, then $q_{i}$ equals 1, and if $\overline{{P_{i} }}$ is smaller than $P_{i}$, then $q_{i}$ equals 0. Therefore, as shown in Fig. 2, if a player has smaller payoff than the average payoff of opponent players, he/she contributes to the punishment pool with high probability. On the other hand, if his/her payoff is nearly equal to the average payoff of opponent players, he/she hardly contributes to the punishment pool. The previous study^37^ reports that the avoidance of overpunishing (too much punishment on defectors with high payoff) is essential for the stable cooperation. In this study, to avoid overpunishing, the payoff of each player will be 0 when it becomes a negative value.

The behaviour of player is updated according to the following rules. That is, each player *i* compares the new payoff $P_{i} ^{\prime}$ with $P_{j} ^{\prime}$ of the players *j* in $O_{i}$ after punishing the opponents and being punished by them. Note that $O_{i}$ represents the set of all players connected to player *i*. Then, each player *i* imitates the behaviour of a player with the highest payoff $max\left( {P_{j} ^{\prime}} \right)$ > $P_{i} ^{\prime}$. If there are multiple players with $max\left( {P_{j} ^{\prime}} \right)$, player *i* randomly imitates the behaviour of the one of such players. If $max\left( {P_{j} ^{\prime}} \right)$ is equal to $P_{i} ^{\prime}$, player *i* randomly switches his/her behaviour to that of the player with $max\left( {P_{j} ^{\prime}} \right)$ including him/her. If $max\left( {P_{j} ^{\prime}} \right)$ is smaller than $P_{i} ^{\prime}$, player *i* does not change his/her behaviour. We consider a series of the public goods game, the process of imposing punishment, and the imitation of behaviour as one generation. One simulation is executed up to 600 generations in order to reach a sufficiently steady state, and we find the average value through 20 times simulations. Table 1 shows the specific parameter settings required for simulations. The values of each parameter conform to Traulsen et al.^12^. Whenever only a single cooperator or defector joins the game, he/she acts as a loner. That is, if only one group member chooses to participate, then all group members receive the loner’s payoff *σ*. The value *σ* = 1 satisfies both conditions *σ* < (*r* − 1)*c*-*G* in the case of pool punishment and *σ* < (*r* − 1)*c* in the case of peer punishment.

**Table 1:** Parameter settings required for simulations.

A finite population (*N*)	1000
The average degree (number of connections) (< *k* >)	4
The average number of players participating in the public goods game (*n*)	5
A factor multiplying all summed-up contributions (*r*)	3
A cost to invest in a common good (*c*)	1
A reduction in the defector payoff for peer punishment (*b*)	1
Punishing a defector cost for peer punishment (*g*)	0.3
A reduction in the defector payoff for pool punishment (*B*)	1
Punishing a defector cost for pool punishment (*G*)	0.3
The loner payoff (*σ*)	1

## Results

**Figure 3:**
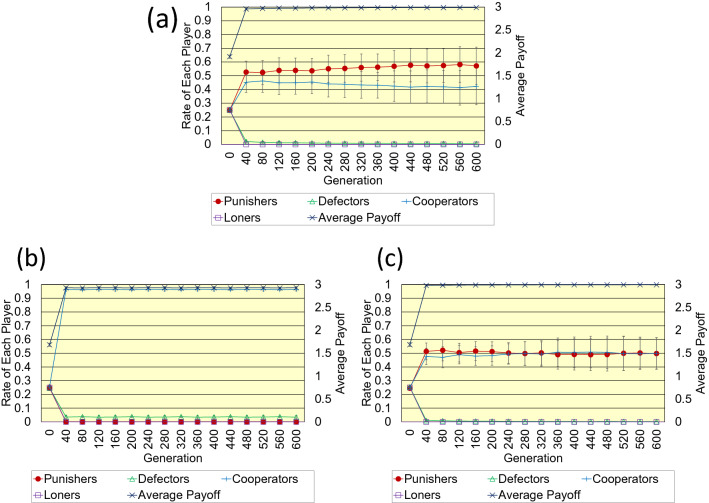
This figure shows the results regarding (**a**) the proposed pool punishment, (**b**) previous pool punishment, and (**c**) previous peer punishment on the regular one-dimensional lattice. Parameter settings are as follows; *N* = 1000, < *k* >  = 4, *n* = 5, *r* = 3, *c* = 1, *b* = 1, *g* = 0.3, *B* = 1, *G* = 0.3, and *σ* = 1.

Below, we compare the proposed pool punishment with previous pool and peer punishment. Firstly, Fig. 3a-c shows the results of the regular one-dimensional lattice. Error bars indicate the standard deviation. (The following figure also has error bars of SD). As those results show, in pool punishment of previous studies, cooperators who do not punish defectors occupy almost the population instead of pool punishers who incur fixed costs. In this case, the average payoff does not reach 3 because a few defectors remain. However, in the proposed pool punishment, more punishers and less cooperators coexist. In peer punishment of previous studies, punishers and cooperators coexist in almost the same number. Those results show that the proposed pool punishment is more robust to the invasion of defectors due to mutation than peer punishment of previous studies. Besides, in terms of the average payoff, the proposed pool punishment is almost the same as previous peer punishment.

**Figure 4:**
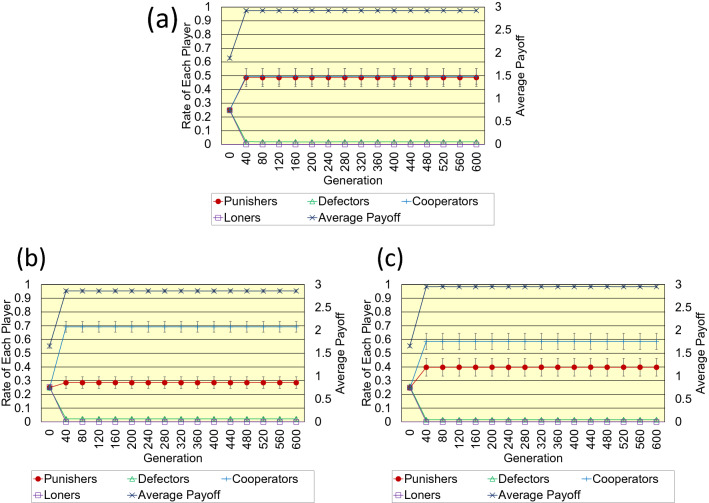
This figure shows the results regarding (**a**) the proposed pool punishment, (**b**) previous pool punishment, and (**c**) previous peer punishment on the random one-dimensional lattice. Parameter settings are as follows; *N* = 1000, < *k* >  = 4, *n* = 5, *r* = 3, *c* = 1, *b* = 1, *g* = 0.3, *B* = 1, *G* = 0.3, and *σ* = 1.

Secondly, Fig. 4a-c shows the results of the random one-dimensional lattice. As those results show, in pool punishment of previous studies, cooperators still dominate, although some punishers remain in the population in comparison with the case of the regular one-dimensional lattice. On the other hand, in the proposed pool punishment, because the payoff of each player is not averaged by his/her number of edges (degree), the difference of payoff between players depending on the degree of each player is larger than in the case of the regular lattice. Therefore, compared to the regular case, the superiority of punishers over cooperators is reduced, and the number of punishers is reduced. Nevertheless, punishers and cooperators coexist in almost the same number, and the robustness against the invasion of defectors due to mutation is maintained because the number of punishers is the largest among three types of punishment. Like the results of the regular lattice, the average payoff of the proposed pool punishment is almost the same as that of previous peer punishment. In previous peer punishment, because the cost of punishment is high, if a punisher has many opponent defectors, those defectors cannot be punished, and then cooperators will have an advantage over such punisher.

The above results show that the proposed pool punishment solves the fixed cost problem of pool punishment of previous studies, and can build a robust state against the invasion of defectors due to mutation. In addition, as the results on the random lattice show, unlike peer punishment of previous studies, punishment is also available due to the low cost of punishment even when a punisher has many opponent defectors.

## Discussion

In the case of the scale-free one-dimensional lattice, which is not mentioned in the results of this study, the author describes what the consequences of the proposed pool punishment and previous pool and peer punishment will be. We compare both cases where the player with the most connections with opponents (the highest degree player) becomes a punisher or a cooperator after the elimination of defectors by punishers. In the case where the highest degree player becomes a punisher, he/she has an advantage over cooperators because he/she can obtain enough payoff to offset the cost of punishment. On the other hand, when he/she becomes a cooperator, he/she has an advantage over punishers because punishers cannot gain enough payoff to offset the cost of punishment. For this reason, in the scale-free one-dimensional lattice, the number of simulations where punishers finally have an advantage and such number where cooperators dominate at last is almost the same regarding the proposed pool punishment and previous pool and peer punishment. This study does not consider punishment on cooperators, the so-called second-order punishment. Therefore, if the number of connections with opponents is the same and the number of punishers or cooperators in opponents is also the same, the highest degree cooperator always has an advantage over punishers in terms of payoff. Considering the second-order punishment, the magnitude relation of payoff between cooperators and punishers naturally changes, then the result can be expected to change.

The author describes the difference between the proposed pool punishment and other probabilistic punishment as follows. Chen et al.^38^ consider probabilistic punishment as the simplest way of distributing the responsibility to sanction defectors. The probability of punishment is fixed among players and does not change depending on the difference of payoff like this study. The following studies also discuss probabilistic punishment: class-specific probabilities of punishment that is based on the fixed number of classes^39^, the implicated punishment that has a working probability *p* (0 < *p* < 1) and includes the peer punishment on defectors with a probability *q* (0 < *q* < 1)^40^. However, those probabilities are fixed and also do not change. Szolnoki and Perc^41^ consider the conditional punishment that does not depend on the difference of payoff like this study, but is proportional to the number of other conditional and unconditional punishers within the group. The proposed pool punishment is similar to Fehr and Schmidt’s inequity aversion^42^ that players resist inequitable outcomes, i.e., they are willing to give up their payoff to realize more equitable outcomes. However, in their study, a player can punish all other players rather than other players having connections with him/her like this study.

Another similar method like the probability of punishment of this study is the emotional profile^43,44^. Szolnoki et al.^43^ introduce sympathy and envy as the two emotional profiles that determine the strategy of each player, and define them as the probability that each player cooperates with players having lower and higher payoff, respectively. The evolutionary process leads to a spontaneous fixation to a single emotional profile; however, this emotional profile depends not only on the payoff but also on the heterogeneity of connections between each player. Szolnoki et al.^44^ also consider the imitation of emotional profiles of neighbour players instead of pure strategy. The emotional profile of each player is determined by two pivotal (not continuous) factors only, namely how each player behaves towards less and more successful neighbour players. On the other hand, the probability of punishment of this study is continuous and based on the difference of payoff.

The following studies, although essentially different, utilize a method similar to the probabilistic pool punishment of this study. Iwasa and Lee^45^ introduce the graduated punishment that the degree of punishment gradually changes based on the damage by selfish behaviour and show that the graduated punishment is the most effective rule in the evolution of cooperation when the action of a player is incorrectly reported at a small probability and the sensitivity of a player to the difference in the utility or payoff is not homogeneous. Helbing et al.^21^ investigate the evolution of cooperation in the spatial public goods game and especially show that increasing the fine of punishment induces a rising of the level of cooperation and larger punishment fines do not have any positive effects. Jiang et al.^46^ also describe that severe punishment is not necessarily more effective and if cooperation is likely, mild punishment leads to higher average payoffs.

This study proposes the probabilistic pool punishment proportional to the difference of payoff in order to eliminate the flaw of pool punishment in which pool punishers incur fixed costs especially if second-order free riders (those who invest in public goods but do not punish defectors) are not punished. In the proposed pool punishment, each player pays the cost to the punishment pool with the probability depending on the difference between his/her payoff and the average payoff of the players with connections to him/her. Comparing the proposed pool punishment with previous pool and peer punishment, in pool punishment of previous studies, cooperators who do not punish defectors become dominant instead of pool punishers who incur fixed costs. However, in the proposed pool punishment, more punishers and less cooperators coexist, and such state is more robust against the invasion of defectors due to mutation than those of previous pool and peer punishment. The average payoff is also comparable to peer punishment of previous studies.

In the future, the author will investigate whether the proposed pool punishment similarly does not allow the invasion of defectors due to mutation and can maintain high average payoff in the cases where second-order free riders are punished^14^, or all types of players can punish other players^5^. The author also intends to devise the probabilistic pool reward and introduce the combination of reward and punishment like the following previous studies^47–49^. Szolnoki and Perc^47^ discuss whether the combined application of reward and punishment is evolutionary advantageous, and find rich dynamical behaviour that shows intricate phase diagrams where continuous and discontinuous phase transitions successively occur. Chen et al.^48^ also propose the institutional sanctioning policy that switches the incentive from rewarding to punishing when the frequency of cooperators exceeds a threshold. They find that this policy establishes and recovers full cooperation at lower cost and under a wider range of conditions than either rewards or penalties alone. Góis et al.^49^ show similar results that rewards (positive incentives) are essential to initiate cooperation and sanctions (negative incentives) are instrumental to maintain cooperation. As each parameter value of this study conforms to Traulsen et al.^12^, a factor multiplying all summed-up contributions (*r*) equals 3, which is relatively large and somewhat induces cooperation. It is also a future work to investigate whether the proposed pool punishment shows good results in the case of low *r* value (e.g. *r* = 2) where cooperation does not easily evolve.

## References

[1] Sigmund K, Hauert C, Nowak MA (2001). Reward and punishment. Proc. Natl. Acad. Sci. U.S.A..

[2] Dreber A, Rand DG, Fudenberg D, Nowak MA (2008). Winners don’t punish. Nature.

[3] Herrmann B, Thöni C, Gächter S (2008). Antisocial punishment across societies. Science.

[4] Ohtsuki H, Iwasa Y, Nowak MA (2009). Indirect reciprocity provides only a narrow margin of efficiency for costly punishment. Nature.

[5] Rand DG, Nowak MA (2011). The evolution of antisocial punishment in optional public goods games. Nat. Commun..

[6] Nowak MA (2006). Five rules for the evolution of cooperation. Science.

[7] Wu J-J, Zhang B-Y, Zhou Z-X, He Q-Q, Zheng X-D, Cressman R, Tao Y (2009). Costly punishment does not always increase cooperation. Proc. Natl. Acad. Sci. U.S.A..

[8] Fehr E, Gächter S (2002). Altruistic punishment in humans. Nature.

[9] Fehr E, Fischbacher U (2003). The nature of human altruism. Nature.

[10] Fowler JH (2005). Altruistic punishment and the origin of cooperation. Proc. Natl. Acad. Sci. U.S.A..

[11] O’Gorman R, Henrich J, Van Vugt M (2009). Constraining free riding in public goods games: Designated solitary punishers can sustain human cooperation. Proc. R. Soc. B.

[12] Traulsen A, Hauert C, De Silva H, Nowak MA, Sigmund K (2009). Exploration dynamics in evolutionary games. Proc. Natl. Acad. Sci. U.S.A..

[13] Rankin DJ, Santos MD, Wedekind C (2009). The evolutionary significance of costly punishment is still to be demonstrated. Proc. Natl. Acad. Sci. U.S.A..

[14] Sigmund K, De Silva H, Traulsen A, Hauert C (2010). Social learning promotes institutions for governing the commons. Nature.

[15] Szolnoki A, Szabó G, Czakó L (2011). Competition of individual and institutional punishments in spatial public goods games. Phys. Rev. E.

[16] Garcia J, Traulsen A (2012). Leaving the loners alone: Evolution of cooperation in the presence of antisocial punishment. J. Theor. Biol..

[17] Perc M, Szolnoki A (2012). Self-organization of punishment in structured populations. New J. Phys..

[18] Nakamaru M, Dieckmann U (2009). Runaway selection for cooperation and strict-and-severe punishment. J. Theor. Biol..

[19] Helbing D, Szolnoki A, Perc M, Szabó G (2010). Evolutionary establishment of moral and double moral standards through spatial interactions. PLoS Comput. Biol..

[20] Szolnoki A, Szabó G, Perc M (2011). Phase diagrams for the spatial public goods game with pool punishment. Phys. Rev. E.

[21] Helbing D, Szolnoki A, Perc M, Szabó G (2010). Punish, but not too hard: How costly punishment spreads in the spatial public goods game. New J. Phys..

[22] Helbing D, Szolnoki A, Perc M, Szabó G (2010). Defector-accelerated cooperativeness and punishment in public goods games with mutations. Phys. Rev. E.

[23] Chen X, Sasaki T, Perc M (2015). Evolution of public cooperation in a monitored society with implicated punishment and within-group enforcement. Sci. Rep..

[24] Sasaki T, Okada I, Uchida S, Chen X (2015). Commitment to cooperation and peer punishment: Its evolution. Games.

[25] Gardner A, West SA (2004). Cooperation and punishment, especially in humans. Am. Nat..

[26] Egas M, Riedl A (2008). The economics of altruistic punishment and the maintenance of cooperation. Proc. R. Soc. B.

[27] Traulsen A, Röhl T, Milinski M (2012). An economic experiment reveals that humans prefer pool punishment to maintain the commons. Proc. R. Soc. B.

[28] Schoenmakers S, Hilbe C, Blasius B, Traulsen A (2014). Sanctions as honest signals—the evolution of pool punishment by public sanctioning institutions. J. Theor. Biol..

[29] Perc M (2012). Sustainable institutionalized punishment requires elimination of second-order free-riders. Sci. Rep..

[30] Chen X, Perc M (2014). Optimal distribution of incentives for public cooperation in heterogeneous interaction environments. Front. Behav. Neurosci..

[31] Perc M, Jordan JJ, Rand DG, Wang Z, Boccaletti S, Szolnoki A (2017). Statistical physics of human cooperation. Phys. Rep..

[32] Ohdaira T (2016). Evolution of cooperation by the introduction of the probabilistic peer-punishment based on the difference of payoff. Sci. Rep..

[33] Ohdaira T (2017). A remarkable effect of the combination of probabilistic peer-punishment and coevolutionary mechanism on the evolution of cooperation. Sci. Rep..

[34] Szolnoki A, Perc M (2017). Second-order free-riding on antisocial punishment restores the effectiveness of prosocial punishment. Phys. Rev. X.

[35] Chen X, Szolnoki A (2018). Punishment and inspection for governing the commons in a feedback-evolving game. PLoS Comput. Biol..

[36] Lee H-W, Cleveland C, Szolnoki A (2022). Mercenary punishment in structured populations. Appl. Math. Comput..

[37] Dercole F, De Carli M, Della Rossa F, Papadopoulos AV (2013). Overpunishing is not necessary to fix cooperation in voluntary public goods games. J. Theor. Biol..

[38] Chen X, Szolnoki A, Perc M (2014). Probabilistic sharing solves the problem of costly punishment. New J. Phys..

[39] Chen X, Szolnoki A, Perc M (2015). Competition and cooperation among different punishing strategies in the spatial public goods game. Phys. Rev. E.

[40] Perc M, Szolnoki A (2015). A double-edged sword: Benefits and pitfalls of heterogeneous punishment in evolutionary inspection games. Sci. Rep..

[41] Szolnoki A, Perc M (2013). Effectiveness of conditional punishment for the evolution of public cooperation. J. Theor. Biol..

[42] Fehr E, Schmidt KM (1999). A theory of fairness, competition, and cooperation. Quart. J. Econ..

[43] Szolnoki A, Xie N-G, Ye Y, Perc M (2013). Evolution of emotions on networks leads to the evolution of cooperation in social dilemmas. Phys. Rev. E.

[44] Szolnoki A, Xie N-G, Wang C, Perc M (2011). Imitating emotions instead of strategies in spatial games elevates social welfare. Europhys. Lett..

[45] Iwasa Y, Lee J-H (2013). Graduated punishment is efficient in resource management if people are heterogeneous. J. Theor. Biol..

[46] Jiang L-L, Perc M, Szolnoki A (2013). If cooperation is likely punish mildly: Insights from economic experiments based on the snowdrift game. PLoS ONE.

[47] Szolnoki A, Perc M (2013). Correlation of positive and negative reciprocity fails to confer an evolutionary advantage: Phase transitions to elementary strategies. Phys. Rev. X.

[48] Chen X, Sasaki T, Brännström Å, Dieckmann U (2014). First carrot, then stick: How the adaptive hybridization of incentives promotes cooperation. J. R. Soc. Interface.

[49] Góis AR, Santos FP, Pacheco JM, Santos FC (2019). Reward and punishment in climate change dilemmas. Sci. Rep..

